# Comment on Brandl et al. Immediate Effects of Myofascial Release on the Thoracolumbar Fascia and Osteopathic Treatment for Acute Low Back Pain on Spine Shape Parameters: A Randomized, Placebo-Controlled Trial. *Life* 2021, *11*, 845

**DOI:** 10.3390/life12060864

**Published:** 2022-06-09

**Authors:** Alexander Leonard Kudus

**Affiliations:** Faculty of Medicine, AMC Hospital, University of Amsterdam, 1100 DD Amsterdam, The Netherlands; alex.kudus@student.uva.nl

I want to congratulate Brandl et al. [1] on their thought-provoking article, entitled “Immediate Effects of Myofascial Release on the Thoracolumbar Fascia and Osteopathic Treatment for Acute Low Back Pain on Spine Shape Parameters: A Randomized, Placebo-Controlled Trial”.

Although very interesting, I do have some reservations after reading the article.

- Brandl et al. hypothesised that myofascial release (MFR) has an effect on the spinal shape in chronic lower back pain (LBP) patients. In their conclusion, there is a significant change between before and after MFR through video raster stereography in functional leg length discrepancy (fLLD) in mm and in degrees of Kyphotic angle.

**Outcome**

**MFR Group**

**OTM Group**

**PLC Group**
fLLD (mm)−5.2−4.5−0.4Kyphotic angle (degrees)8.32−8.42−0.8Lordotic angle (degrees)1.5−5.00.1

At baseline, they measure the pain using the visual analogue scale (VAS). There is no mention of remeasuring the VAS after the treatment. Thus, what is the clinical implication of a 5.2 mm improvement fLLD and 8 degrees of change in the kyphotic angle when disregarding the pain VAS? 

The video raster stereography (VRS) has moderate accuracy in measuring scoliosis degree and low accuracy in monitoring curve progression, according to T. Bassani et al. [2]. The average difference between radiography (RAD) and VRS is 18 degrees of uncertainty. The within-subjects correlation is 0.3 according to Bassani et al. [2]. Brandl et al. [1] refer to the accuracy of the study of Degenhardt et al. [3] with a small set of 30 subjects.

T. Bassani [2] comparted the VRS accuracy with 192 subjects.

Taken this into account, we can question the meaningfulness of the significant VRS kyphotic outcome difference of 8 degrees in this study.

In my opinion, we need more qualitative research to build a good scientific foundation for MFR treatments.
